# Consumer Law and Policy Relating to Change of Circumstances Due to the COVID-19 Pandemic

**DOI:** 10.1007/s10603-020-09463-z

**Published:** 2020-08-03

**Authors:** COVID-19- Consumer Law Research Group, Richard Alderman, Alberto De Franceschi, Mark Giancaspro, Geraint Howells, Chen Lei, Javier Lete, Hans-W Micklitz, Emilia Miscenic, Tjakie Naude, Pascal Pichonnaz, Elise Poillot, Iain Ramsay, Evelyne Terryn, Christian Twigg-Flesner, Thomas Wilhelmsson

**Affiliations:** School of Law, University of Warwick, Warwick, CV4 7AL UK

**Keywords:** Consumer law, COVID-19, Moratoria, Refunds, Guiding principles

## Abstract

An unprecedented number of consumer problems has been caused by the COVID-19 pandemic, not least with regard to refunds of prepayments and the ability of consumers to keep up their monthly payments under loan and rental agreements. Based on a notion of *societal force majeure* sketched in this paper, we propose guiding principles in respect of the introduction of moratoria on recurring payments, the use of refunds or vouchers in respect of prepayments, and associated enforcement challenges. This analysis draws on experiences around the globe.

The COVID-19 pandemic has swept around the world. As well as huge suffering and high numbers of fatalities, it has had a heavy negative economic impact. Millions of consumers around the world are suffering financially, because many have lost their regular income, and many are fighting to obtain refunds of prepayments from struggling businesses whilst having to service loans and rental payments. In this contribution, we, a research group on consumer law with a global reach, offer an initial assessment of the legal difficulties consumers face. We argue for a principles-based way forward and evaluate a range of global initiatives taken thus far.

## Common Consumer Problems Due to the Pandemic

In addition to widespread loss of life and human suffering, COVID-19 is impacting on a wide range of societal activities, often with significant legal consequences. Consumer issues have been at the forefront during the pandemic. Examples of price gouging have led some countries to introduce price controls (e.g., Croatia, Italy, and South Africa). Unscrupulous traders have exploited health concerns by making unwarranted claims for COVID-19-related products or have supplied counterfeit or of poor quality products. Our focus is, however, on how the changed circumstances due to COVID-19 impact on established contractual relations. In particular, we look at two important areas of concern due to the COVID-19 pandemic:

(i) Consumer debt
(ii) Consumers seeking refunds for cancelled travel bookings and events

These problems not only affect substantial numbers of consumers but are also forcing radical thinking to find just and manageable solutions. Moratoria on consumer credit and/or rent obligations have been introduced, and the idea of debt jubilees has been raised. Travel companies are struggling to reimburse the large number of cancellations on time and are sometimes seeking to give consumers vouchers or credits instead of a refund to assist their own liquidity and ensure their survival. These are novel challenges facing legal systems, and they seem poorly equipped to handle them using their traditional legal tools. Some states have responded with tailored legislative responses to COVID-19, but these often raise as many questions as they answer. We seek to provide some principles to guide the legal response to contracts affected by COVID-19 and to demonstrate how these might apply in the two areas considered. In drafting and applying these principles, it should be recognized that vulnerable consumers will be amongst the worst affected by the pandemic and are likely to be unable to bear the financial impact to the same extent as the majority of citizens. Also, states will find themselves able to support citizens and businesses to different degrees with many developing countries only able to provide minimal support. Specific rights call therefore also for effective, and potentially collective, solutions.

## Existing Legal Regimes

Contracts can contain provisions specifying what should happen when unforeseen circumstances arise. In some legal systems, this may be subject to a good faith provision to prevent abuse of rights. Traders may plausibly argue that the scale of the pandemic takes it outside any potential scenario envisaged and thereby justify renegotiation of existing contracts. Equally, in consumer contracts, any term that is imbalanced against consumers may be subject to challenge under laws controlling the use of unfair contract terms.

Absent express clauses, legal systems have a limited number of tools to seek to achieve justice in changed circumstances. One traditional criticism of legal rules on *force majeure* or impossibility or, in common law systems, the doctrine of frustration, is that they set a high threshold for invoking them as these generally require that performance has become impossible. For COVID-19-related cases, this will often not be a barrier as legislation enforcing lockdowns has made many consumer contracts impossible or unduly burdensome to perform. Potentially, it may be an issue when a contract is suspended for only part of its duration, as in the case of an annual gym membership or contract for a live sports channel, or where only limited services are offered. Such rules have also traditionally, in most legal systems, failed to take adequate account of life events (illness, redundancy, etc.) that impact on the consumer’s ability to perform the contract. Furthermore, the remedies are typically rather blunt and not well suited to fashion solutions for the situations generated by COVID-19.

The common law knows of no obligation to renegotiate. Either the contract is frustrated or the original obligations remain in place. Many civil law systems use the principles of good faith and *rebus* sic *stantibus* to impose a duty to renegotiate in extraordinary circumstances, and to allow the courts to impose solutions where agreement cannot be reached. However, there is little guidance as to what renegotiated contract terms should be imposed in response to COVID-19-induced change of circumstances. There are also laws governing specific sectors that provide for tailored solutions. In Europe, the Package Travel Directive is one notable example of such legislation.

A problem with all these traditional private law solutions is that they rely on individualized renegotiation or litigation. This is unrealistic in the COVID-19 context as the volume of claims would fall like an avalanche on the legal systems.

## Actions Taken in Response to the Pandemic

Several states have taken specific action to address the pandemic problem. Some, like Germany, have introduced moratoria on consumer debts or, like South Africa, have introduced restrictions on the enforcement of debts. In the United Kingdom (UK), similar results have been achieved by the financial regulator, the Financial Conduct Authority (FCA), fine-tuning their rules. The period of moratorium varies typically between three and six months. However, there remains uncertainty about the consequences of the moratorium and what happens when it ends.

Belgium and Italy are two countries that have introduced legislation that permits, subject to certain conditions, vouchers to be given in respect of cancelled events or package travel holidays. Although the South African Consumer Goods and Services Ombud has acknowledged consumers’ right to a refund, she tried to persuade consumers to accept vouchers where this was possible and, somewhat confusingly, ends a press release by suggesting that suppliers may not be liable for a refund after all if a consumer unreasonably refuses the supplier’s offer to supply comparable goods or services later (Consumer Goods and Services Ombud 2020).

The problems raised by the pandemic are crying out for a principled approach. The European Law Institute has made a start by setting out *Principles for the Covid-19 Crisis* with principle 12 on moratorium of regular payments and principle 13 on force majeure and hardship being of relevance (ELI 2020). However, the consumer context raises particular issues we consider merit more specific guidance and an approach that balances the interests of consumers and traders in these unique circumstances.

## Principles for Responding to the Pandemic

All private law regimes have some doctrine concerning the impact of changed circumstances. These have various contents and use various labels. However, what they can offer is not enough with regard to the challenges raised by the pandemic for several reasons.

The impact of the pandemic is felt on an unprecedented scale for both businesses and consumers. As a global crisis, and a crisis that affects all branches of society and economy, it goes far beyond what the traditional legal means were developed for. It is not the context for which consumer law, and general private law, was designed. In addition to issues on a micro-level of individual contracts, there are simultaneously macro-level issues of a more systemic nature. The latter include a need for relatively quick solutions, to ensure that money is paid or reimbursed rapidly, to avoid further insolvency issues, as well as a need to prevent as much as possible that courts are paralysed with several thousand additional COVID-19-related cases. The collapse of Lehman Brothers in 2008 and the Euro crisis which followed taught us that consumer debts have an impact on the economy at large. In those instances, consumers were the debtors. The pandemic has added a new complication: Consumers are turning into creditors of the tourism and leisure industry, where their individual rights for refunds are not being honoured. We argue that a solution has to be guided, at least to some extent, by new principles.

### A Dynamic and Cooperative Understanding of Contract

First, as the impact of the pandemic is felt everywhere, the number of potential legal disputes related to the pandemic is both globally and nationally overwhelming. This makes the use of traditional legal means related to changed circumstances impractical, as they often heavily rely on an assessment of the individual case, ultimately by courts. Potentially new procedural means should be fostered, as presented below. They need, however, to be driven by a more relational understanding of contract. The idea of co-operation is much more developed in long-term contracts. Even in the case of spot contracts, matters do not end with the initial execution of the contract either—spot contracts also have a post-contractual phase. This issue has gained fresh significance due to the pandemic. Therefore, the principle of *pacta sunt servanda* should be conceived in a more dynamic and cooperative way, imposing a duty on parties to collaborate in finding creative solutions to keep the contractual relationship and parties afloat, and sharing in a fair way the consequences of the pandemic.

Much of the impact of the crisis affects all parties to the contractual chains involved. Many actors are suffering. Therefore, a traditional private law on/off-solution might not be the best instrument in such cases, since it might affect randomly parties in the contractual chain, without an understanding of the overall perspective. Compromise solutions should be agreed both in bringing to the table as many parties of the contractual chain involved as possible, and in distributing the burden between the parties in a fair way. For example, in landlord and tenant relations, one should not only envisage moratoria (“breathing spaces”) or longer periods of time to pay the rent (see below), but in some instances provide for a fair reduction of the rent for a certain period. In consumer lease contracts, such a fair reduction may be triggered by the fact that tenants have lost their jobs or had to accept reduced salaries due to partial unemployment related to COVID-19 measures.

### Social and Societal Force Majeure

For consumers, the situation can often be classified as a situation of *social force majeure* (Wilhelmsson 1992, pp. 180–216), protecting consumers who, as a result of unforeseeable developments, have been hit by unemployment or illness. Here, there are lessons to learn from the Euro crisis. Those consumers who were most in need of support were not the ones who benefitted from national support programmes aimed at banking liquidity at that time. Quite the contrary seemed to be true. Consumer law needs to develop principles on how to deal with conflicting needs, similar to the discourse on intersectionality in the context of discrimination law, by seeking to identify those who are in greatest need of support.

As a kind of “extraordinary contract law” (Pichonnaz 2020), the search for co-operative solutions entails displacing the more rigid contractual or legal devices for allocating unforeseen risks and changed circumstances, when dealing with the contractual consequences of the measures related to the COVID-19 pandemic. The present principles better reflect the collective impact of the pandemic on all the involved interests, not only consumers but also businesses. They should seek to balance the impact on the whole of the economy, rather than just protecting one player, be it a business, be it a consumer, or more specifically be it business sectors and particular types of consumers. A principle of “*societal* force majeure” could recognize that not just consumers are financially impacted, but that also businesses are in severe and unexpected financial difficulties.

### Incentives to Encourage Cooperative Solutions

A case-by-case basis is inappropriate; the current situation requires an approach that combines individual and collective remedies, not only for procedural reasons, but also to prevent strong parties in a contractual relationship from abusing their power during individual negotiations, either because of their contractual power, or their ability to allocate their risk on more contracts, or even because of their better knowledge of the effects of some measures taken by the authorities. The answer to these threats is, however, not only procedural, by relying on collective or sectorial negotiations or further means (see below). It should also go to the substance. For example, authorities should not inject huge amounts of money to sustain travel agencies and airline companies without requiring them to give their contractual parties a real and effective right to get a fair refund of their advance payments.

### Incentives to Encourage Sustainable Solutions

An approach that combines individual and collective remedies and seeks to balance the impact on the whole economy also creates opportunities to favour those solutions that facilitate the transition towards more sustainable consumption and production (Sarkis et al. 2020). In terms of sustainable consumption, the current crisis has triggered behavioural changes with both negative (such as increased use of packaging, substituting private for public transport) and positive effects (such as the increased uptake of shorter producer-to-consumer models, less (unnecessary) commuting) (Boons et al. 2020). In the search for cooperative solutions to this crisis, the sustainability aspect can and must be taken into account. Thus, for example, when renegotiating public transport subscriptions, an extension of the contract term could be combined with more flexibility to allow for continued teleworking.

## Response 1: Moratoria

The COVID-19 pandemic significantly affects rights and obligations of both creditors and consumers as contracting parties to credit agreements across the world. Due to loss or decrease of income, consumers face serious financial difficulties resulting in their inability to repay credit instalments on a monthly basis. Moreover, the COVID-19 pandemic is forcing some consumers to overcome the poor financial situation by entering into new consumer credit agreements, often at higher interest rates. Bearing in mind the devastating economic consequences that could result from the significant imbalance in contracting parties’ rights and obligations caused by the pandemic, many jurisdictions reacted with the so-called “moratorium” proposals suspending or postponing consumers’ credit payment obligation from three to six months (Act of 20 May 2020 on Consumer Credit, to Help Borrowers Combat the Crisis Caused by the Coronavirus (Belgium); Act to Mitigate the Consequences of the COVID-19 Pandemic under Civil, Insolvency and Criminal Procedure (Germany); Royal Decree-Law on Urgent Special Measures To Tackle the Economic and Social Impact of COVID-19 (Spain); Ordonnance COVID-19 bail à loyer et bail à ferme (Switzerland)). In some instances, the obligation has not been suspended, but a moratorium has been placed on enforcement of any default. For example, in the United States of America (USA), there is a moratorium on payment of federally backed student loans for 180 days, but no rules on private student loans (CARES Act, sec. 4022).

A federal moratorium exists on evictions (but not on the payment of rent) for tenants in certain types of properties backed by federally protected mortgages, but only limited state-by-state legal protections for other tenants (CARES Act, sec. 4024(b)) and a state-by-state limit on collection efforts, such as garnishment and lawsuits, for consumer debt (e.g., Texas Supreme Court, Emergency Order 15 - Issued 05/14/2020). England and Wales paused possession actions for private rentals for 3 months (Coronavirus Act 2020, s.81 and Schedule 29) but provided no moratorium on rental payments. These legislative and non-legislative measures often deal only partially, or not at all, with a variety of issues related to consumer loans.^1^

Below, we identify the most important issues affecting contracting parties to consumer loans, namely the question of interest rates during and after the moratorium, the impact on credit ratings and the length of the moratorium. We also propose measures for protection of mortgage and rent payers.

### Interest Rates During and After Moratorium

Many moratoria permit interest to continue to run during the moratorium period (Financial Conduct Authority 2020a, b). There are examples of legal solutions, where demanding payment of any additional contractual costs in form of fees or interest is not allowed (e.g., in Belgium). This is the rule in the US for federally backed student loans, which suspends interest and does not allow fees or late charges.

There might be concerns that loan contracts are renegotiated in a manner that banks are “paid” for a moratorium, e.g., through additional fees or charges. Such commercial behaviour could in the future be challenged in a number of individual and collective consumer redress proceedings. On the other hand, due to mandatory arbitration agreements prohibiting collective redress in most financial agreements, this probably is not an option in the USA. Despite the amendments being presented as an “option” to consumers, the contractual terms on “paid” moratoriums could be challenged under EU law using the *unfairness test* (Directive 93/13/EEC, Art. 3(1)). However, such contractual terms could be qualified by national courts as essential elements of the contract if they are affecting the amount of the loan’s principal (as main subject matter) and interest (as price). In that case, contractual terms would be excluded from the unfairness test if they satisfy the so-called transparency requirement (Miscenic 2018, p.143). In contrast, EBA guidelines do not consider adaptation of variable interest rate during moratorium as an amendment of the contract (EBA 2020).

In the light of foregoing and bearing in mind that “moratorium” means suspension, postponement or reducing of party’s obligations, we suggest that during the moratorium period, requiring payment of additional contractual costs in form of fees or interest should not be allowed. This legal solution is accepted in Spain when the borrower is under a situation of economic vulnerability according to the requirements of the new regulation.

### Prolongation of Contract Duration

Since the pandemic is affecting both sides of the contractual relationship and is unlikely to have run its course within the three-month period of most current moratoria, we argue that the costs incurred by the creditor are best mitigated by extending the credit contract duration. Consumer credit agreements should either be extended for the time period of the “moratorium” and for several months after, or a compromise reduction agreed in the obligations of the parties. Such a legal solution would ensure the returning to balance of the contracting parties’ rights and obligations during the extended period of time. The prolonged contractual period would extend the instalments payments under a credit agreement and give consumer-borrowers the opportunity to adapt their financial situation to new life circumstances and the new economic situation on the labour market whilst ensuring a greater likelihood of ultimate repayment. This proposal should not in any case affect other consumer rights under the loans contract, such as the right to early repayment of a loan.

As an example, Section 4022 of US CARES Act provides relief to student loan borrowers during the COVID-19 national emergency. Federal student loan borrowers are automatically being placed in an administrative forbearance, which sets interest at 0% and allows borrowers to temporarily stop making monthly loan payments. This suspension of payments and interest will last until September 30, 2020. Borrowers may still make payments, which will be applied to the principal.

### Impact on Creditworthiness Assessment

Although, the issue is extensively regulated by EU Directives on consumer credit (Directive 2008/48/EC, Art. 8; Directive 2014/17/ЕU, Arts. 4(17) and 18-21), norms of anti-discrimination, and data protection, the conditions affecting the “credit scoring” are partly regulated by national credit laws or are determined by internal criteria and assessment of credit institutions. These criteria usually encompass the personal and economic status of the debtor, liquidity and assets, debtor’s income and prospects on the employment market, as well as exposure to interest, exchange and other financial risks. This gives credit institutions leeway to adjust their rules on creditworthiness assessments that should be adequately adapted to the circumstances of the credit market during the moratorium stage. Bearing in mind the effects of the moratorium and our proposed legal solution on extending contracts, we propose postponement of creditworthiness assessments during the moratorium, and, after the moratorium has expired, preventing adverse inferences in respect of a consumer’s creditworthiness if they have benefitted from a moratorium.^2^ For instance, Belgian law provides explicitly that the suspension may not be recorded in the central credit registry as a default (Act of 20 May 2020 on Consumer Credit, to Help Borrowers Combat the Crisis Caused by the Coronavirus).

### Prevention of Foreclosure Proceedings and Evictions

Closely related to the moratorium of contractual rights and obligations under consumer credit agreements are the use of foreclosure in the case of mortgages or eviction by landlords. According to the current extraordinary laws of many states, enforcement proceedings have, in principle, been stayed during the time period of three–six months, depending upon the respective country (Act to Mitigate the Consequences of the COVID-19 Pandemic under Civil, Insolvency and Criminal Procedure Law (Germany)). For example, in the USA, there is a six-month federal moratorium on evictions for tenants in certain types of properties backed by federally protected mortgages, but only limited state-by-state legal protections for other tenants (CARES Act, sec. 4024(b)). However, once that moratorium is over, evictions could continue and eventually cause a lot of both economic and personal damage to consumers, who will lose their homes and assets due to their inability of repay the debts. The purpose of Spanish legislation on moratorium in mortgage and other credit loans, and also for renters, is to guarantee the right to housing for borrowers in a situation of special vulnerability, whose income has been reduced as a consequence of health crisis (see preamble of the Royal Decree-Law of 17 March 2020).

The prolongation of the contract period should be used by both parties to re-establish a proper balance between contracting parties’ rights and obligations. Moreover, the importance of the ‘fundamental right to a home’ must be taken into account as protected under ECHR and EU Charter of Fundamental Rights. A United Nations Guidance Note suggests considering moratoria on evictions and rent payments, and even “rent and mortgage forgiveness for particularly vulnerable households” (United Nations 2020, no. 8). Equally, under Directive 2014/17/EU, Art. 28, EU Member States should encourage creditors to exercise reasonable forbearance before initiating foreclosure proceedings, and ensure that there are measures to facilitate repayment of consumer debts (FCA no date A, 7.3.17; FCA no date B, 13.3.2A(c)).

## Response 2: Refunds and Vouchers

### Business Cancellation or Substantial Delay

A very disruptive element of a crisis such as the pandemic is the cancellation or substantial delay of a pre-paid event, or services, due to restrictions imposed by state or local government. Weddings, sports events, cruises, air-travel, concerts, and similar events are planned in advance and it is not easy, or sometimes even possible, to reschedule. Businesses may lack sufficient resources to issue immediate refunds, and such an obligation may cause it to lay off employees, exacerbating the number of financially vulnerable consumers.

Three alternative approaches present themselves. First, consumers may be entitled to a full refund, or at *their option* a voucher for future use, if the event is cancelled or substantially delayed. This is currently the rule in several countries, and many airlines, with respect to airfares (Regulation (EC) 261/2004; Cathay Pacific 2020; US Department of Transportation 2020). In favour of such a policy is the consideration that the economic burden is often substantially greater for the consumer.

To encourage consumers to choose a voucher for future use, it is suggested providers offer a “bonus amount,” such as 15–25%, should the consumer agree to receive a voucher instead of a refund. This is currently the policy for some companies, e.g., Singapore Airlines (2020). If a voucher is issued, it should be valid for at least 18 months from the date of issue, regardless of the contract’s terms. The Australian Competition and Consumer Commission (ACCC) has stipulated that any credit vouchers or notes issued should have an expiry date “long enough” to enable consumers to make use of the voucher (ACCC 2020). The European Commission has recommended a minimum validity of 12 months. Also, vouchers may be made “attractive” by allowing consumers to use them for any services offered by the provider (or other entities of the same group), or by making them transferable to other travellers without additional costs (European Commission 2020).

A second possible approach is to have a special dispensation for a crisis such as the pandemic, obliging consumers to accept vouchers, subject to some safeguards. Because many pre-planned events are purchased at a substantial discount, any “voucher” offered by the business must be for the same class of ticket or service for the future event, even if the cost has risen. Safeguards regarding recreational events in Belgium are (i) the voucher must have the same essential characteristics; (ii) the event will take place within two years of the original event date; (iii) the voucher represents the entire price of the ticket, no surcharges; and (iv) the voucher explicitly mentions the COVID-19 crisis (Ministerieel besluit betreffende de privé- en publieke activiteiten van culturele, maatschappelijke, festieve, folkloristische, sportieve en recreatieve aard 2020). A similar solution has been adopted in Italy with the following safeguards: (i) buyers must have claimed for reimbursement within 30 days from 19 May 2020 or from the communication of the impossibility of performance; (ii) the event will take place within 18 months of the original event date, and (iii) the voucher represents the entire price of the ticket (Law Decree on urgent measures regarding health, sustain to labour and economy, as well as social policies connected with the epidemiological emergency 2020).

Other safeguards may be that vouchers have a cash-in value at a later point, and that there be some protection if the business goes insolvent, e.g., by government.

The third, intermediate, approach, again for specific application in the context of a crisis, is to require consumers to accept vouchers equal to the value of their original ticket, unless the consumer can show that they are in financial difficulties, e.g., due to being furloughed or unemployed, or are otherwise unable to take up the service later. Otherwise, to cancel, the consumer must pay the cancellation fees subject to the original contract terms.

Which solution is best will probably vary across legal systems. Countries of the global south, such as many African countries, have considerably fewer resources than developed ones to help prevent businesses from going insolvent, and for social security to assist the unemployed, which may have devastating effects such as hunger leading to social unrest and crime. The refund option may be far harsher on businesses, their employees and society in general in these countries and so it is suggested that the third option may be more appropriate there. The African principle of *ubuntu*, which stresses human interdependence, co-responsibility, and concern (*S v Makwanyane*^1995^, para.224), may further justify such an outcome in African countries with low resources. However, in more developed countries, it is proposed that the refund option coupled with strong incentives to choose vouchers instead is the best one. We recognize requiring a refund imposes an economic toll on the service provider, however, and we assume some negotiation regarding refund or voucher will take place.

A related issue is the effect of the pandemic on consumer rights with respect to services. For example, the Australian Consumer Law requires businesses supplying services to guarantee that those services are appropriately rendered ‘fit for purpose’ (Competition and Consumer Act 2010, sch 2, s 61), while businesses are also prohibited from receiving payment for services they do not intend to supply. For example, a river cruise company’s failure to provide tour services of an enjoyable nature due to high river levels was deemed to contravene this provision (*Moore v Scenic Tours Pty Limited*^2017^). It is conceivable consumers impacted by COVID-19 could make similar arguments regarding affected travel plans. This situation may also arise where, for example, a gymnasium continued charging subscribed members under their membership contracts knowing their facilities cannot be operating while under restriction.

### Consumer Cancellation or Rescheduling

A more difficult situation arises when the event is not directly adversely affected by the pandemic, but rather by the consumer’s concerns and fears regarding the safety of attending or conducting the event. Examples might include a scheduled cruise that is to set sail months after restrictions have been put in place due to the pandemic, a scheduled event is available after limits on large groups are lifted, or a flight is scheduled after restrictions are ended. Consumers may still feel unsafe to travel or hold the event at that time.

In such cases, the remedy should be a refund or travel voucher in the amount paid for future use, but at *the option* of the provider. If the consumer insists on a refund, the contract terms or other applicable law should control, unless the terms are found to be unfair or unconscionable. Some countries’ existing legislation may allow cancellation of all bookings, subject to payment of a reasonable cancellation penalty, e.g., in South Africa (Consumer Protection Act 2008, section 17).

We argue that all parties should act reasonably and fairly and work together to minimize the impact of the decision to provide a voucher, but recognize that the business may incur costs due to the rescheduling. In such cases, contract terms regarding fees for rescheduling should be considered, and negotiated. Due to the nature of the pandemic and legitimate fears regarding resuming normal activities, providers should also consider extending the time period during which events may be rescheduled to 18 months, even in cases where contracts provide for a shorter period.

## Resolution

To address some of the more pressing issues, states have sometimes adopted emergency regulations imposing solutions to solve some of the issues linked to consumer credit or mortgage, to commercial leases or, sometimes even, to ordinary private leases, as well as for package travel and other transport contracts that have been cancelled. These legislative solutions may be coupled with administrative sanctions in case of infringement, but often they do not provide for an effective remedy to implement the solutions provided for in the regulation. There is a pervasive idea that parties will mostly be willing to follow the regulations. Given the extraordinary economic pressure both on professionals and consumers, it is however far from sure that parties will be ready or even able to follow the solutions.

The principles of *social* and *societal* force majeure as presented above may call for more co-operative solutions. The scope and impact of the crisis may, however, also affect the way these cooperative solutions are arrived at and eventually enforced. The sheer number of cases linked to both individual cooperative solutions and enforcement of regulatory solutions may well overwhelm both courts and ADR systems, if cases are handled in an individualized way. Those bodies might well not be able to deliver the swift decisions that are required to avoid further insolvencies and harmful economic adverse situations.

A more collective resolution mechanism is therefore also needed. Although the collective dimension is not novel to consumer law, the effective resolution of mass cases remains a weakness both in many national systems and at EU level, notwithstanding the various recent initiatives that were taken in this regard (European Commission 2018). Even in national jurisdictions where collective actions (or even class actions) are possible, the adversarial character, complexity, and potential length of the proceedings may stand in the way of the swift and balanced outcome sought for in the aftermath of the COVID-19 crisis. However, their procedures may also provide possibilities to come to a negotiated solution that can then be declared binding on all consumers involved by the court (thus, e.g., the WCAM system in the Netherlands that allows to declare a settlement binding on an opt-out basis and the Belgian class actions Act that has an obligatory negotiation phase before the (adversarial) part procedure can be continued).

Such collective negotiations may however also take place outside of a formal class actions system. One could envisage sectorial negotiations between national representatives of specific businesses and national or regional consumer protection organizations. These sectorial negotiations would be monitored by a COVID-19 mediator, who could, for example, be appointed by the Ministry of Trade in given countries. The COVID-19 mediator would be aware of other similar negotiations, so the creative models that may be found in one area might be adapted or reproduced in other. The result of such collective negotiations could then be implemented by parties, instead of having to seek individual solutions (unless doing so would result in a more suitable outcome). In the absence of consensual implementation, each party would then be able to ask for quick and efficient enforcement (Pichonnaz 2020, pp. 150–153). This combination of attempting voluntary solutions backed by effective enforcement as a fall-back could be effectively implemented, leaving enough room for individual parties to find more appropriate solutions when this is possible. A negotiated solution may also enhance the readiness of parties voluntarily to enforce those solutions, without an intervention of a public enforcement body.

In the absence of voluntary compliance, the additional powers granted in recent years to some public enforcers, allowing them to negotiate or impose a solution that includes redress or compensation for consumers and to accept commitments in case of an infringement of consumer law, may be a welcome tool to deal with the aftermath of the COVID-19 crisis. Good examples in this regard are the powers of the UK Financial Conduct Authority to impose consumer redress schemes (Financial Services and Markets Act 2000, s.404) as well as the UK’s Consumer Rights Act (2015) that allows a broad range of public enforcers to decide on a case by case basis what is the best way to deal with an infringement (Department for Business, Innovation, and Skills 2015, p. 7). At EU level, the revised CPC regulation requires member states to ensure that national consumer authorities have the power to seek or accept commitments in case of an infringement of consumer law (Regulation (EU) 2017/2394, Art. 8).

## Conclusion

One lesson we can learn from the impact of the COVID-19 pandemic on consumers is that many of our traditional consumer protection rules are ill-suited to deal with the circumstances consumers around the world now face. Our focus on moratoria and refunds shows that national governments and regulators are confronting similar problems but are experimenting with different ways of addressing these concerns. The rapid adoption of new rules often lacks a sound principled basis, and the after-effects of these measures, once lifted, tend to be as-yet unaddressed. Furthermore, the differential impact of the crisis on consumers and the associated challenges for consumers to enforce their rights will necessitate a much more thorough review of dispute resolution and enforcement mechanisms. Our discussion seeks to provide guidance to legislators and regulators around the world for moving forward.

## Footnotes

1 The European Banking Authority (EBA) has issued guidelines (EBA 2020) for banks to clarify when a moratorium does not constitute forbearance under existing rules (defined as a “concession by an institution towards an obligor that is experiencing or is likely to experience difficulties in meeting its financial commitments” (Regulation (EU) 575/2013, Art.47b(1))).

2 This view corresponds to EBA guidelines which emphasize that moratorium has to be available to a large, predefined group of borrowers, “regardless of the assessment of their creditworthiness” (EBA Guidelines, nos. 20 and 27).
